# Overview of Allelopathic Potential of *Lemna minor* L. Obtained from a Shallow Eutrophic Lake

**DOI:** 10.3390/molecules27113428

**Published:** 2022-05-26

**Authors:** Julia Gostyńska, Radosław Pankiewicz, Zdzisława Romanowska-Duda, Beata Messyasz

**Affiliations:** 1 Department of Hydrobiology, Faculty of Biology, Adam Mickiewicz University in Poznan, Uniwersytetu Poznanskiego 6, 61-614 Poznan, Poland; julgos@amu.edu.pl; 2 Faculty of Chemistry, Adam Mickiewicz University in Poznan, Uniwersytetu Poznanskiego 8, 61-614 Poznan, Poland; radpan@amu.edu.pl; 3 Department of Plant Ecophysiology, Faculty of Biology and Environmental Protection, University of Lodz, Banacha 12/16, 90-237 Lodz, Poland; zdzislawa.romanowska@biol.uni.lodz.pl

**Keywords:** duckweed, pleustophytes, macroalgae, allelopathy, competition, polyphenols, population formation, eutrophy

## Abstract

Allelopathy is an interaction that releases allelochemicals (chemicals that act allelopathically) from plants into the environment that can limit or stimulate the development, reproduction, and survival of target organisms and alter the environment. *Lemna minor* L. contains chemicals that are allelopathic, such as phenolic acids. Chemical compounds contained in *L. minor* may have a significant impact on the development and the rate of multiplication and lead to stronger competition, which may enhance the allelopathic potential. Allelopathic potential may exist between *L. minor* and *C. glomerata* (L) Kütz. because they occupy a similar space in the aquatic ecosystem, have a similar preference for the amount of light, and compete for similar habitat resources. *L. minor* and *C. glomerata* can form dense populations on the water surface. Allelopathy can be seen as a wish to dominate one of the plants in the aquatic ecosystem. By creating a place for the development of extensive mats, an interspecific interaction is created and one of the species achieves competitive success. It is most effective as a result of the release of chemicals by macrophytes into the aquatic environment. Therefore, allelopathy plays a significant role in the formation, stabilization, and dynamics of the structure of plant communities.

## 1. Introduction

The plant community is shaped by specific plant species that coexist in a particular place at a particular time [1]. Their presence in a community is determined by many ecological aspects, including biotic and abiotic factors [2]. Interspecies relationships are an important biotic factor, as a result of which individuals compete with each other. These interactions take place directly or indirectly in the individual–individual and individual–environment relationships [1]. As a consequence, it is possible to inhibit the development of one of the co-occurring species, which ensures the competitive success of another species. One example of such an interaction is allelopathy. Allelopathy is a series of interactions that occur in the environment between various organisms [3]. As a result of this process, mixtures of allelopathic chemicals are released from plants into the environment [3]. Allelochemicals can directly or indirectly limit or stimulate the development, reproduction, and survival of target organisms, also affecting the environment [4].

### 1.1. Allelopathic Potential of Lemna minor *L.*

Allelopathy can have a variety of practical applications in both terrestrial and aquatic environments. However, it is most commonly used in agriculture, e.g., to remove weeds, diseases, and microorganisms that are harmful to crops, as well as to improve the condition of crops and to effectively increase their yields [5,6,7]. Allelopathy is probably one of the most modern and effective methods for agriculture. In turn, allelopathic substances act in a similar way to herbicides, being an effective tool for controlling weeds in plant crops [8]. According to research, *Lemna minor* L. contains chemicals that are allelopathic [9,10]. Previous studies have confirmed that aqueous methanol extracts isolated from *L. minor* have a significant effect on monocotyledons (cress, lettuce, and alfalfa) and dicotyledons (rye grass, timothy, barnyard grass, crab grass, and junglerice) [5]. Small amounts of methanol extract (0.1 g DW eq. extract mL^−1^) limit the growth of shoots and roots in cress, lettuce, and rye grass, as well as timothy and crab grass roots [5]. At a higher concentration (1 g DW eq. extract mL^−1^), the development of shoots and roots in barnyard grass, junglerice, crab grass, and rye grass is inhibited [5]. Among the plants studied, methanol extract most strongly inhibited the development of alfalfa shoots and timothy roots [5]. Subsequent studies have shown that substances present in *L. minor*, such as flavonoids and fatty acids, have an inhibitory effect on the growth of walnut biomass [5]. As reported in the literature, plant extracts are also bacteriostatic in nature, which has been demonstrated in *Sphaerotilus natans* [7]. In *L. minor*, there are substances with antioxidant and anti-radical properties and pharmacological properties, such as phytol; campesterol; loliolide; dihydroactinediol; ascorbic acid; vanillic acid; 2,3-dihydroxybenzoic acid; caffeic acid; chlorogenic acid; esculetin; esculin; and fraxetin [10,11,12]. A characteristic feature of *L. minor* is its simple morphology, rapid biomass growth rate, and high sensitivity to changes in the ecosystem. As a result, it is often used in toxicological studies of the environment [8,9,13]. Because of its high protein content, *Lemna minor* is used in agriculture as an effective biopesticide [5,6,13]. It eliminates phenolic compounds from the aquatic environment, which arise as a result of industrial development and are toxic to organisms [13,14]. This confirms the bioremediation abilities of *L. minor* [14]. Bioremediation consists in improving the ecological condition of the environment with the use of organisms and their ability to remove or reduce the concentration of harmful pollutants. Due to the fact that *L. minor* is resistant to oxidative stress and the accumulation of reactive oxygen species (ROS), which are the result of the presence of phenolic compounds in the environment, it may contribute to the improvement of water quality [13,15]. The ability to produce biomass quickly can be used to treat wastewater with a high content of organic compounds or heavy metals. *L. minor* has a high tolerance to water contaminated with metals [16]. It is a good hyperaccumulator of lead, nickel, chromium, copper, cadmium, and manganese [16]. This plant is also highly effective in removing arsenic, Ni, Zn, Fe, and Cd from aquatic ecosystems [16]. So far, little research has been carried out to confirm the allelopathic potential of *Lemna minor* or to explain the mechanism of this phenomenon. Nevertheless, the use of this plant as a tool for managing the aquatic and terrestrial environment seems to be an extremely important solution for polluted ecosystems.

### 1.2. Models of Lemna minor *L.* and Cladophora glomerata (*L.*) Kütz Population Formation: Interactions between Species

Large amounts of nutrients are present in eutrophic water bodies. The consequence of this is the excessive development of biological life, including the massive appearance of phytoplankton organisms and other aquatic plants that create blooms on the water surface and reduce the transparency of the water. An excessive development of green algae, mainly *Cladophora* (Chlorophyta) species, is observed in eutrophic water reservoirs. *Lemna minor*, belonging to the Lemnaceae family, is also a common aquatic plant.

*Lemna minor* is a small plant (pleustophyte) with a simple morphological structure. It consists of a single thallus and has no root [17,18]. The plant reproduces mainly vegetatively [19]. In summer, *L. minor* forms single-species, dense, and compact clusters that float freely on the water surface [9,20]. These patches inhibit the development of other macrophytes, limiting their access to light by up to 99% [21]. The increase in plant biomass over a short time contributes to the eutrophication of the aquatic ecosystem. To survive the colder months, the pleustophyte forms small turions that are filled with starch and sink to the bottom of the water reservoir [10,22]. Most often, *L. minor* occurs in waters with a temperature of 6 to 33 °C. It also has a wide range of pH tolerance, from 5 to 9 [23]. *L. minor* prefers shallow places with low water turbulence that are rich in electrolytes [21,24]. The growth, survival rate, and growth rate are influenced by various ecological factors, e.g., pH, water temperature, concentration of nutrients, presence and concentration of toxins in water, as well as competition with other plants for light and nutrients [22]. However, temperature and light availability are the factors that best stimulate the proper growth and development of *L. minor* [22]. It has the ability to absorb minerals as well as phosphorus and potassium that are present in nutrient-rich water [22]. A threat to the development of *L. minor* is the intense growth of algae, including the green algae of the genus *Cladophora*, which limit the plant’s access to light and nutrients [22]. As a result, it has limited space and resources to create dense colonies on the surface of a body of water.

*Cladophora glomerata* (L) Kütz. is a green alga that dominates in eutrophic waters [25,26]. Due to the diversified morphology of the plant, the thallus may be long and branched in flowing waters and short and bushy in stagnant waters [27]. The lifting force of water enables the movement of the thallus, which can change its shape and place of occurrence. It creates filamentous forms, called grippers, by means of which it attaches to the ground or floats in the water column [27]. *C. glomerata* forms dense and large populations (“mats”) on the surface of a water body [21,28]. Intensive development occurs in spring and autumn, as a result of which it creates massive blooms [27]. The density and size of the biomass often depend on the growing season and the environmental conditions in which it grows [29]. Green alga mats may consist of individuals of one species (*Cladophora glomerata*) or of several co-occurring species [25]. Although *C. glomerata* occurs in most types of aquatic ecosystems (from stagnant waters to flowing waters), it is most numerous in eutrophic waters with a high concentration of nitrogen and phosphorus [21,25]. It has preference for a wide range of temperatures and light conditions; therefore, it easily adapts to the ecosystem it currently inhabits [21,25]. Owing to its uncomplicated morphological structure, rapid multiplication rate, and high ecological tolerance, *C. glomerata* easily forms large populations and occupies considerable space [26,29]. This alga can limit access to light by up to 70%, create anaerobic conditions, and inhibit the supply of nutrients and thus inhibit the development of macrophytes [26,29,30]. *C. glomerata*, similarly to *L. minor*, prefers waters with a high content of nutrients as well as nitrogen and phosphorus [29]. An important element for the proper growth of green algae is the optimal chemical composition of water, in which there is a high concentration of nitrates and orthophosphates [25].

As shown in the above literature data, *L. minor* and *C. glomerata* are species with similar ecological preferences. Both plants occupy a similar space in the aquatic ecosystem, have a similar preference for the amount of light, and compete for similar habitat resources. Both *L. minor* and *C. glomerata* can form dense populations that float on the water surface. As a result, they inhibit the growth of other hydro macrophytes as they restrict their access to light and nutrients. Their chemical composition indicates the presence of substances with an allelopathic effect. The presence of phenols such as phenyl ester, methoxylphenol, coumaric acid, and benzoic acid has been found in the thalli of filamentous green algae [31]. Unfortunately, there is little research explaining the mechanism of interaction between these plant species. However, it can be concluded that there is an allelopathy phenomenon between them, which may be visible when one plant wants to dominate the aquatic ecosystem. Creating a place for the development of extensive mats allows an interspecific interaction (allelopathy) to occur, thanks to which one of the species achieves competitive success. This is an important point that we want to highlight in this article. Therefore, this paper provides an overview of the allelopathic potential of duckweed and reviews the studies on models of *Lemna minor* and *Cladophora glomerata* population formation with a focus on their chemical composition. Moreover, with reference to the studies, the aim of the research presented in the paper is to answer the following questions:

1. Do *Lemna minor* and *Cladophora glomerata* secrete allelopathic substances in coexistence?
2. Does the allelopathic potential occur in competing interactions?

## 2. Chemical Composition of *L. minor* and *C. glomerata*

According to the data contained in the literature, the cell wall of *Lemna minor* consists of: carbohydrates (51.2%), dry starch (19.9%), cellulose, and pectins (20.3%), along with galacturonan, xylogalacturonan, rhamnogalacturonan, and hemicellulose (3.5%), with xyloglucan and xylan and phenols (0.03%) [10]. The chemical composition indicates that in the dry matter of *L. minor*, there are: proteins (up to 35%), fibers (up to 17%), fats (up to 5%), polysaccharides, flavonoids, amino acids, aliphatic compounds, phenolic acids, triterpenes, micro- and macronutrients, vitamins (A, B, and E), and carotenoids [10,11,32]. Among fatty acids, the most numerous are polyunsaturated fatty acids (PUFA), which constitute 60-63% of all fatty acids [33]. Among these acids, the highest amounts are found in α-linolenic acid (41–47%) and linoleic acid (17–18%) [33]; α-linolenic acid, linoleic acid, and palmitic acid also constitute 80% of all fatty acids [10]. In terms of chemical composition, the plant further includes unsaturated acids (76.7%), mainly oleic and linoleic, and saturated acids (23.3%), including mainly palmitic and stearic acids [10]. *L. minor* contains essential amino acids (39.20%), non-essential amino acids (53.64%), and non-proteinogenic amino acids (7.13%) [10]. Their content is presented in Table 1. The following are dominant in essential amino acids: leucine, isoleucine, and valine (48.67%). In non-essential amino acids, glutamic acid (25.87%) is dominant [10]. The chemical composition also includes citrulline, hydroxyproline, taurine, histidine, leucine, lysine, methionine, phenylalanine, threonine, and tryptophan [10], while the lipophilic substances are: hexanal; trans-2-heptenal; caproic acid; eticaproate; trans-2-octenal; ethylheptanoate; nonanal; 2,6-dimethylcyclohexanol; menthol; pyrrole-2,5-dione; tetradecane; pentadecane; dihydroactinediol; heptadecane; loliolide; ethyltetradecanoate; trans-neophytadiene; hexahydrofarnesylactone; cis-neophytadiene; ethyl pentadecanoate; ethyl palmitate; heneicosan; phytol; tricosan; pentacosan; heptacosan; spinesterol; stigmasterol; sitosterol; and campesterol [10,11].

In *L. minor*, there are also substances with antioxidant activity, such as: phytol; campesterol; loliolide; dihydroactinediolide; ascorbic acid; vanillic acid; 2,3-dihydroxybenzoic acid; caffeic acid; chlorogenic acid; esculetin; esculin; and fraxetin [10,11]. The content of the chemical elements present in the plant is shown in Table 2 [10,11,34]. The literature emphasizes the importance of the above-mentioned chemical compounds, mainly in the regulation of plant growth and development. Glutamic acid plays an important role in many physiological processes of plants, responsible for seed germination, stimulation of plant growth and development, dying, response to environmental stress, and adaptation to changing ecological conditions [35]. It is an amino acid precursor. It plays a significant role in nitrogen storage and transport, increasing nitrogen absorption in plants, including *L. minor* [36]. Valine is responsible for the effective functioning of the immune system in stressful situations for plants. Isoleucine is an essential substrate for protein synthesis, and leucine is responsible for the synthesis of phytohormones [33]. Unsaturated fatty acids play a major role in the proper functioning of defense systems against stress factors [37]. This suggests that the chemical compounds contained in *L. minor* may have a significant impact on appropriate development and lead to a faster multiplication rate and stronger competition, which may strengthen the allelopathic potential.

Considering the chemical composition, *C. glomerata* is composed of carbohydrates, proteins, lipids, and other chemical compounds (Table 3). The most common minerals in this species are: potassium, calcium, iron, aluminum, magnesium, sodium, and chrome (Table 4). Among these elements, the highest dry matter content is that of potassium (94.1 g·kg^−1^), followed by those of calcium (56.4 g·kg^−1^), iron (26.5 g·kg^−1^), aluminum (23.1 g·kg^−1^), magnesium (13.5 g·kg^−1^), sodium (11.5 g·kg^−1^), and chrome (0.247 g·kg^−1^). Literature data show that in terms of chemical composition, *C. glomerata* also includes: n-hexadecanoic acid; mono (2-ethylhexyl) ester; tetradecanoic acid; 2-pentadecanone; 6,10,14-trimethyl; tetradecanoic acid; 12-methyl ester; hexadecanoic acid; ethyl ester; and 9,12 octadecadio-neoyl chloridae (Table 5).

## 3. Results

### 3.1. Chemical Composition Analysis of L. minor and C. glomerata

The analysis of the chemical composition of *L. minor* and *C. glomerata* showed the content of chemical elements (Table 6 and Table 7). In the case of *L. minor*, the following elements were identified: potassium, calcium, phosphorus, magnesium, nitrogen, iron, boron, copper, zinc, and manganese (Table 6). Among all analyzed elements, potassium (2194 mg·kg^−1^) and calcium (1,3020 mg·kg^−1^) were characterized by the highest content in *L. minor* compared to other elements. Manganese (68.4 mg·kg^−1^) had the lowest amount in *L. minor*. In the case of *C. glomerata*, the following elements were identified: calcium, potassium, magnesium, lead, arsenic, sodium, iron, nickel, manganese, cadmium, copper, zinc, cobalt, and chrome (Table 7). The highest content was that of calcium (148.2 ± 3.1) in dry mass. Chrome had the lowest content (0.01 ± 0.001) in dry mass.

### 3.2. Analysis of the Morphological Features of Cladophora glomerata and Lemna minor

**Figure 1:**
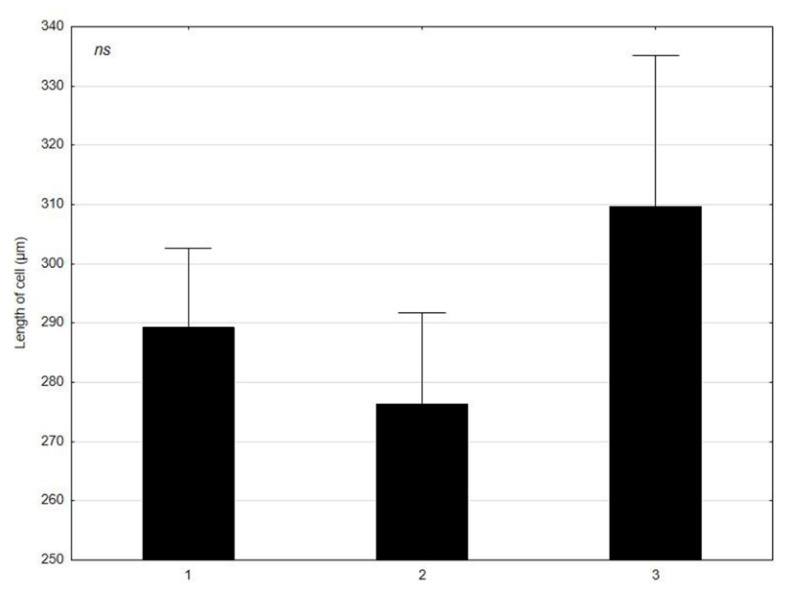
Mean values of *C. glomerata* feature (length of cells) at different times (1–30 April 2021; 2–4 June 2021; 3–17 July 2021) presented using the Tukey multiple comparisons test. The bars indicate mean values. Error bars represent standard error. A statistically significant result for *p* ≤ 0.05 was marked.

**Figure 2:**
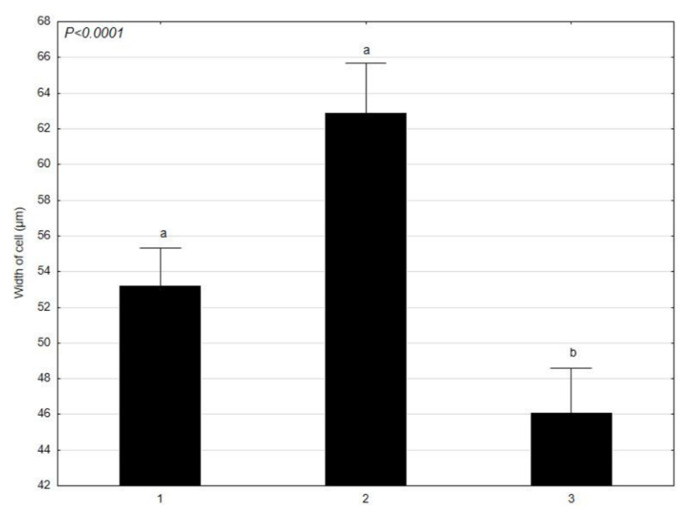
Mean values of *C. glomerata* feature (width of cells) at different times (1–30 April 2021; 2–4 June 2021; 3–17 July 2021) presented using the Tukey multiple comparisons test. The bars indicate mean values. Error bars represent standard error. A, B, C determines the statistical significance between the analyzed features. A statistically significant result for *p* ≤ 0.05 was marked.

**Figure 3:**
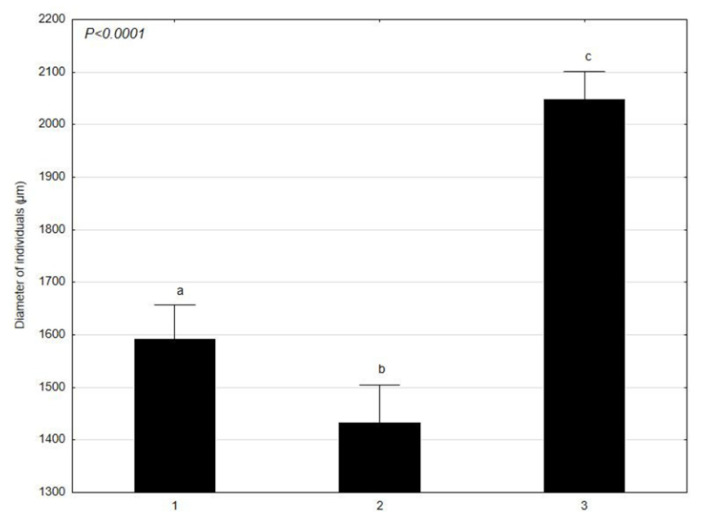
Mean values of *L. minor* feature (diameter of individuals) at different times (1–30 April 2021; 2–4 June 2021; 3–17 July 2021) presented using the Tukey multiple comparisons test. The bars indicate mean values. Error bars represent standard error. A, B, C determines the statistical significance between the analyzed features. A statistically significant result for *p* ≤ 0.05 was marked.

**Figure 4:**
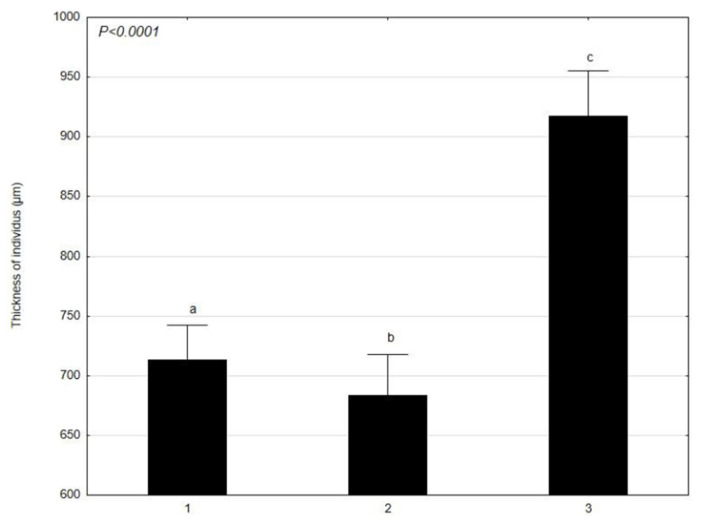
Mean values of *L. minor* feature (thickness of individuals) at different times (1–30 April 2021; 2–4 June 2021; 3–17 July 2021) presented using the Tukey multiple comparisons test. The bars indicate mean values. Error bars represent standard error. A, B, C determines the statistical significance between the analyzed features. A statistically significant result for *p* ≤ 0.05 was marked.

*C. glomerata* was analyzed in terms of the mean values of the morphological features, such as: the length and width of cells at various times (1–30 April 2021; 2–4 June 2021; 3–17 July 2021) (Figure 1 and Figure 2). *L. minor* was analyzed in terms of the mean values of the morphological features, such as: the diameter and thickness of individuals at various times (1–30 April 2021; 2–4 June 2021; 3–17 July 2021) (Figure 3 and Figure 4). The test sample contained 30 *C. glomerata* cells and 30 *L. minor* individuals. Cell length values in *C. glomerata* were not statistically significant. The *C. glomerata* cell width was statistically significant in April (a) and June (a) in relation to the cell width in July (b). Statistically significant differences were also observed in the diameters and thicknesses of *L. minor* cells. The diameters of *L. minor* cells showed statistically significant differences among themselves (a, b, c) in April, June, and July. Cell thicknesses also showed significant differences between themselves (a, b, c) in April, June, and July. The highest mean cell length (310 μm) of *C. glomerata* was in July and the lowest in June (276.5 μm). The greatest difference was between the average widths in June (63 μm) and July (46.1 μm). In the case of *L. minor*, the highest average diameter (2048.6 μm) and thickness (918 μm) of individuals was in July. The smallest average diameter (1434.6 μm) and thickness (684.5 μm) of individuals was in June.

### 3.3. The Size of the Patches of L. minor and C. glomerata in the Littoral Zone

The mean value, the minimum value, the maximum value, and the standard deviation were analyzed in terms of the size of the patches of *C. glomerata* and *L. minor* in the littoral zone (Table 8). The size of the patches was analyzed on three different dates: 30 April 2021; 4 June 2021 and 17 July 2021. The mean value, the minimum value, the maximum value, and the standard deviation of *Cladophora glomerata* showed that the smallest minimum (0.01 m^3^) size of the *C. glomerata* patch occurred on 30 April 2021 and the largest minimal (0.65 m^3^) occurred on 17 July 2021. The smallest maximum (0.02 m^3^) size of a patch of this species occurred on 30 April 2021 and the largest maximum (0.76 m^3^) on 17 July 2021. The smallest value of the standard deviation was 0.005 m^3^ (30 April 2021). The highest standard deviation value was 0.055678 m^3^ (17 July 2021). The size of *L. minor* patches differed from that of *C. glomerata*. The smallest minimum size of an *L. minor* plot was 0.1 m^3^, which occurred on 30 April 2021 and 4 June 2021. The largest minimum size of a plot of this species was, however, 0.08 m^3^ (17 July 2021). The smallest maximum value of the *L. minor* plot was 0.1 m^3^ (17 July 2021) and the highest 0.19 m^3^ (4 June 2021). The smallest average value of the patch was 0.09 m^3^ and occurred on 17 July 2021 and the highest was 0.15 m^3^ and occurred on 30 April 2021 and 4 June 2021. The smallest standard deviation was 0.01 m^3^ (17 July 2021) and the largest amounted to 0.05 m^3^ (30 April 2021).

### 3.4. The Content of Phenolic Acids in L. minor and C. glomerata

Phenolic acids were found in all the tested *C. glomerata* individuals from Oporzyńskie Lake, *L. minor* individuals from Oporzyńskie Lake, and *L. minor* from breeding (Table 9). The phenol content in *C. glomerata* was significantly higher than the phenol content in *L. minor.* Table 4 shows that the lowest minimum concentration of phenols (7.418 mg GAE g^−1^) in *C. glomerata* occurred on 30 April 2021 and the highest minimum concentration of phenols (17.934 mg GAE g^−1^) was on 4 June 2021. The lowest maximum value of phenol concentration was 7.601 mg GAE g^−1^ (30 April 2021), and the highest maximum was 18.459 mg GAE g^−1^ (4 June 2021). The lowest average concentration of phenols was 7.515 mg GAE g^−1^, which occurred on 30 April 2021. The highest average concentration of phenols was 18.209 mg GAE g^−1^, which occurred on 4 June 2021. The concentration of phenols in *L. minor* collected from Oporzyńskie Lake significantly differed from the concentration of phenols in individuals from the farm and from the concentration of phenols in *C. glomerata*. The lowest minimum concentration of phenols in the species *L. minor* from Oporzyńskie Lake was 2.111 mg GAE g^−1^ (30 April 2021), and the highest minimum concentration of phenols was 9.423 mg GAE g^−1^ (17 July 2021). The lowest minimum concentration of phenols was 2.892 mg GAE g^−1^ (30 April 2021) and the highest 9.816 mg GAE g^−1^ (17 July 2021). The lowest average concentration of phenols was also 2.111 mg GAE g^−1^ (30 April 2021), and the highest was 9.582 mg GAE g^−1^ (17 July 2021). The lowest minimal (1.794 mg GAE g^−1^), the lowest maximum (2.006 mg GAE g^−1^), and average (1.902 mg GAE g^−1^) concentrations of phenols in individuals from the cultivation of *L. minor* occurred on 30 April 2021. The minimum concentration of phenols (2.029 mg GAE g^−1^) occurred on 17 July 2021 and the highest maximum (2.052 mg GAE g^−1^) and the highest mean were recorded on 4 June 2021.

### 3.5. Relationships between the Parameters of Dry Mass of L. minor and C. glomerata and the Effect of Polyphenol Concentration and Time

**Figure 5:**
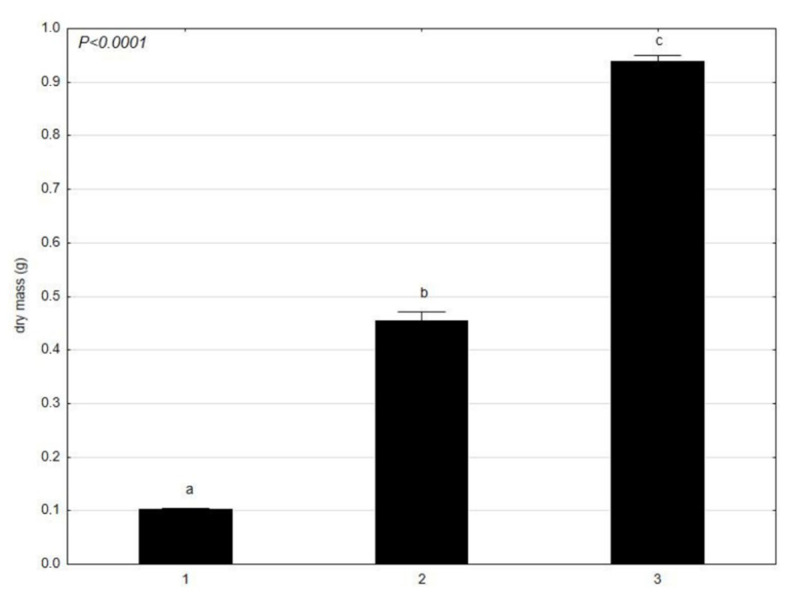
Mean value of dry mass (g) of *C. glomerata* at different times (1–30 April 2021; 2–4 June 2021; 3–17 July 2021) presented using the Tukey multiple comparisons test. The bars indicate mean values. Error bars represent standard error. A, B, C determines the statistical significance between the analyzed features. A statistically significant result for *p* ≤ 0.05 was marked.

**Figure 6:**
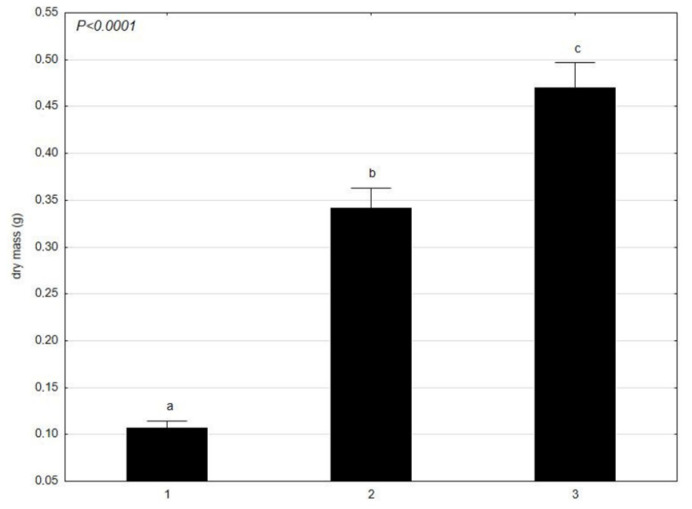
Mean value of dry mass (g) of *L. minor* in different times (1–30 April 2021; 2–4 June 2021; 3–17 July 2021) presented using the Tukey multiple comparisons test. The bars indicate mean values. Error bars represent standard error. A, B, C determines the statistical significance between the analyzed features. A statistically significant result for *p* ≤ 0.05 was marked.

The results of the dry mass content in *C. glomerata* (Figure 5) and L. minor (Figure 6) showed that *C. glomerata* had a higher dry mass content in June and July than *L. minor*. In April, *C. glomerata* had a slightly lower content of dry mass than *L. minor*. Considering the results separately, the dry mass of *C. glomerata* had the highest mean value in July and the lowest in April. It was similar for L. minor—the highest average dry mass value was in July and the lowest in April. Both in *C. glomerata* and *L. minor*, the mean value of dry matter was statistically significant between the tested trials from April, June, and July (a, b, c).

**Figure 7:**
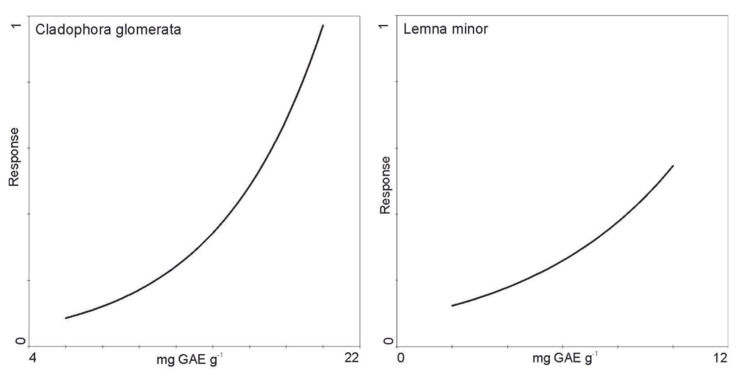
*C. glomerata* and *L. minor* response curves to time of polyphenol gradient modeled by the generalized additive model using Poisson distribution smooth term complexity selected according to the AIC criterion. The first axis represents the GAE, and the second axis represents the dry mass.

**Figure 8:**
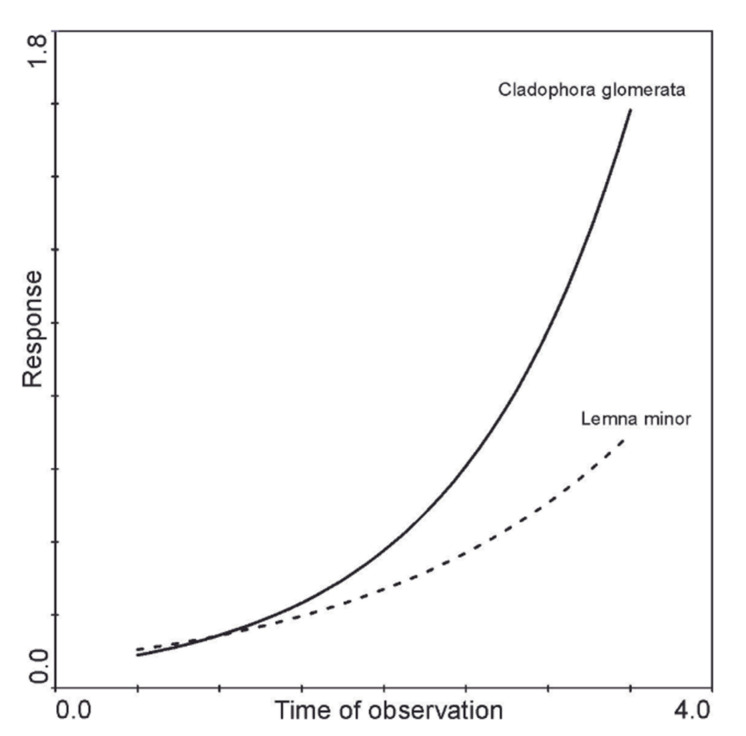
*C. glomerata* and *L. minor* response curves to time of observation (4 month) gradient modeled by the generalized additive model using Poisson distribution smooth term complexity selected according to the AIC criterion. The first axis represents the time values, and the second axis represents the dry mass.

The response of *C. glomerata* and *L. minor* to the polyphenol content (Figure 7) and the effect of the observation time (Figure 8) were analyzed using the GAM model. The first model showed that in both *C. glomerata* and *L. minor*, the concentration of polyphenols increased in cells over time (Figure 7). However, in the case of *C. glomerata*, the biomass increased faster with increasing polyphenol concentration (Figure 7). The biomass of *L. minor* increased significantly slower with the increase in the concentration of polyphenols as compared to *C. glomerata* (Figure 7). The second model showed that *C. glomerata* is characterized by a faster increase in biomass during the observation than *L. minor* (Figure 8).

## 4. Discussion

Taxa co-occurring in the same place may use allelopathy as a strategy in competition for space, nutrients, light, and other ecological factors [26,40]. Competition can result in the replacement or exclusion of a species. It is most effective as a result of the release of chemicals by hydro macrophytes into the aquatic environment. Therefore, allelopathy plays a significant role in the formation, stabilization, and dynamics of the structure of plant communities [6,26,41]. Both *Lemna minor* and *Cladophora glomerata* inhabit common habitats, show similar preferences for the availability of light and nutrients, and create dense mats on the water surface. Plants need space for proper development, so they compete with each other for the surface layer of water.

The conducted analyses showed that: (1) *Lemna minor* and *Cladophora glomerata* secrete allelopathic substances, (2) allelochemicals have a significant impact on the structure of pleustophyte and alga communities, and (3) allelopathic potential occurs as a result of competition between co-occurring species.

The results of our research show that the average length and width of cells in *C. glomerata* as well as the diameter and thickness of *L. minor* individuals showed significant differences in the analyzed time (April–June–July). The highest mean length of *C. glomerata* cells was in July and the lowest in June. In the case of *L. minor*, the highest average diameter and thickness of individuals was in July (in the fullest of the growing season). The smallest average diameter and thickness of individuals was in June. Considering the size of the patches of *C. glomerata* in the littoral zone, the largest occurred in July and the smallest in April. *L. minor*, however, had the largest patches in April and the smallest in July. Additionally, these results show that *C. glomerata* created more extensive patches on the water surface of Oporzyńskie Lake as compared to *L. minor*.

The length and width of *C. glomerata* cells and *L. minor* individuals as well as the size of their patches (“mats”) also showed a relationship with the total phenolic content in these species. It is worth noting that the phenol content in *C. glomerata* was higher than the phenol content in *L. minor*. The highest concentration of phenols in green algae had the highest and most similar values in June and July and the lowest in April. *L. minor* that was collected from Oporzyńskie Lake had the highest concentration of phenols in July and the lowest in April. *L. minor* from the cultivation had a lower content of phenols compared to *L. minor* and *C. glomerata* that were collected from the Oporzyńskie Lake. In this case, phenols reached their highest values in June and lowest in April.

These results show that both *C. glomerata and L. minor* have allelopathic potential. However, *C. glomerata* shows greater potential and therefore is a stronger taxon in interspecies competition. *C. glomerata* has a specific feature. This is the rapid development of the thallus and dynamic multiplication. At the same time, it produces a large quantity of phenolic compounds. Our research confirms these features. Both in *C. glomerata* and in *L*. *minor*, the concentration of polyphenols in the cells increases with time. However, the biomass of *C. glomerata* grows much faster compared to that of *L. minor*. As a result, *C. glomerata* can form broad and dense mats on the water surface and successfully compete for space with duckweed. The macroalga *C. glomerata* characteristically dominates in eutrophic waters [25] and has a rapid biomass growth over time, and our findings have confirmed this dependence. As a result, the alga excludes the co-occurring taxon and achieves competitive success over other aquatic plants [24,25]. This mechanism provides green algae with a large space for proper development and access to light and nutrients. Phenolic compounds secreted by *C. glomerata* may influence the formation of communities of small pleustophytes, including *L. minor*. *C. glomerata* showed a higher total phenolic content than *L. minor*. The greater the concentration of green algae in the surface water layers, the more phenols are released into the environment. This inhibits the development of *L. minor* and limits the size of its patches on the water surface. The strategy of freshwater algae in the process of competition is: intensive development in early spring, taking nutrients from the water, chemical composition of the thallus (presence of proline in the period of high thallus density), changes in the morphological structure, secretion of allelopathic substances, and the formation of spores [26] (from Ozimek 1990).

*Lemna minor* may also inhibit the development of other hydromacrophytes. This pleustophyte creates dense mats on the water surface. As a result, it reduces the amount of light available for the deeper layers of water, which is necessary for other plants to grow adequately [20]. Both the literature data and our results show that this pleustophyte contains allelopathic chemicals. During coexistence with *C. glomerata*, a higher content of phenolic compounds was demonstrated in *L. minor* individuals than in bred individuals. This shows that *L. minor* also has a competitive strategy. Pleustophyte competes with *C. glomerata* for space, light, and nutrients, so it uses its allelopathic potential to achieve competitive success. When bred, *L. minor* does not have to compete with other plants for space and access to nutrients. Therefore, the total phenolic content is lower as it is not necessary for survival. It is likely that these allelochemicals may also inhibit the development of other plants. As a result, *L. minor* can ensure a competitive advantage in the aquatic ecosystem [20]. However, the mechanism of the allelopathic strategy has not yet been elucidated.

## 5. Conclusions

Finally, we would emphasize that our efforts have been directed in two ways: (i) to review the studies on models of *Lemna minor* and *Cladophora glomerata* population formation with a focus on their chemical composition and (ii) to mark a particular model of seasonal growth of two species occupying one ecological niche at the same time. In our study, the concentration of polyphenols in the cells of *C. glomerata* and in *L*. *minor* increases with time. The role of rapid biomass growth of one of the competing species seems to be crucial. To assess the effectiveness of *Lemna minor* allelopathic potential, many factors need to be determined. The research carried out in this article highlights the poorly understood relationships between species (duckweed and macroalga) that coexist in the same place at the same time in the aquatic environment. The mechanisms that would accurately explain the phenomenon of allelopathy between competing taxa are also unknown. These issues require further research, which we will certainly undertake in well-defined aquatic systems during laboratory experiments.

## 6. Materials and Methods

### 6.1. Raw Material Collection and Identification

Freshwater *Cladophora glomerata* thalli and *Lemna minor* specimens were collected from the shallow Lake Oporzyńskie (N 52°55′70″, E 17°09′60″), situated in the northern part of the Wielkopolska region (western Poland), in the April–July period of 2021, when the algal biomass was at its annual minimum and maximum. Characterization of physical and chemical parameters during the intensive development of *C. glomerata* in the lake has been performed earlier [24,25,42]. Morphometric measurements of the length and width of cells and individuals were taken using a light microscope (LM).

### 6.2. Element Analysis of Lemna minor and Cladophora glomerata Raw Material

The content of selected elements, such as Ca, Mg, Na, K, Fe, Zn, Cu, As, Cd, Ni, Pb, Cr, Mn, and Co, was determined in the *Lemna* and *Cladophora* biomass. The elemental analysis was carried out in an inductively coupled plasma–optical emission spectrometer (Varian ICP-OES VISTA-MPX) by the ICP–OES method. The concentration of elements was expressed as µg∙g^−1^ of dry matter or g∙g^−1^ of dry matter or mg∙kg^−1^ (in the case of *Lemna minor*). Then the analysis error was calculated.

### 6.3. Ultrasound-Assisted Extraction (UAE)

Ultrasound-assisted extracts were made out of raw, powdered material of *C. glomerata* and *L. minor*. In each case, for the preparation of extracts for spectrometric analysis, 10 g of dry weight of material was extracted in an ultrasonic bath with two portions of methanol as a solvent (2 × 100 mL), over a total time of 1 h. After 30 min, the first portion of the solvent was removed and a new portion was added to continue the extraction for another 30 min. The temperature of the ultrasonic bath did not exceed 35 °C. The extracts were filtrated, and the filtrates were combined. The solvent was removed in the rotary evaporator. To prepare samples for spectrometric analysis, the methanolic solutions of extracts were made up to a concentration of 10.00 ± 0.06 mg mL^−1^.

### 6.4. Determination of Total Phenolic Compounds in Extracts

A calibration curve was prepared by dissolving gallic acid in 70% methanol to obtain a stock solution with a concentration of 1 mg/mL, after which subsequent dilutions were made in a range of concentrations from 0.1 mg/mL to 1 mg/mL. Then 20 µL of gallic acid solution with a particular concentration was added, along with 1.58 mL of distilled water, 0.1 mL of Folin–Ciocalteu reagent, and 0.3 mL of saturated solution of sodium bicarbonate (Na2CO3). The final reaction volume was 2 mL, and the final concentration of gallic acid ranged between 0.001 and 0.01 mg/mL [43]. A reaction mixture with real samples was prepared in the same way as the samples for the calibration curve, and the extract solution was added instead of gallic acid solution. The result was expressed as gallic acid equivalent (GAE) using the equation C [mg QE/gextract] = C [mg/mL] × (V1 [mL]/V2 [mL]) × (V3 [mL]/m [g]), where C (mg/mL) is the concentration from the calibration curve, V1 is the total volume of the reaction vessel, V2 is the volume of the extract/standard added to the reaction, V3 is the volume in which the extract was dissolved, and m is the mass of the extract dissolved in V3 to prepare a real sample of extract. After keeping the reaction vessels in darkness for 2 h, the samples were measured with a UV/VIS spectrometer at 760 nm. Each sample was prepared and measured in triplicate. Data are the mean ± the SD values.

### 6.5. Data Analysis

Statistical analyses were performed with STATISTICA software version 12.0. To confirm the significance of differences between the analyzed *Lemna minor* and *Cladophora glomerata* features in time, a one-way ANOVA followed by Tukey’s RIR post hoc test was used. Differences were considered to be significant at *p* < 0.05. These statistical analyses were performed using the R 3.0.1 statistical package (R Development Core Team 2013, using the vegan package; [44]).

Changes of *Lemna* and *Cladophora* biomass occurrence in response to time were modeled using the GAMs [45]. We used Poisson distribution, while smooth term complexity was selected using the Akaike information criterion [46].

## Figures and Tables

**Table 1:** Amino acid content in *L. minor* [10].

The Name of the Amino Acid	Amino Acid Amount (%)
Glutamic acid	13.53
Leucine	10.27
Aspartic acid	9.89
Alanine	7.88
Valine	7.67
Glycine	7.36
Phenylalanine	6.28
Lysine	6.2
Isoleucine	5.89
Threonine	5.08
Proline	4.88
Arginine	4.67
Serine	4.05
Tyrosine	2.76
Histidine	2.32
Tryptophan	0.85
Methionine	0.39
Cystine	Small amounts

**Table 2:** The content of chemical elements in *L. minor*.

Name Chemical Element	The Amount of Chemical Element (mg/100 g) [10,11]	Dry Mass (%) [34]
Calcium	4990	0.18
Potassium	2495	1.53
Silicon	2495	-
Sodium	1870	0.02
Manganese	935	0.03
Iron	934	0.06
Phosphorus	515	0.83
Magnesium	155	1.92
Aluminum	0.93	-
Nickel	0.93	-
Copper	0.78	-
Lead	0.03	-
Molybdenum	0.02	-
Zinc	0.01	0.05
Nitrogen	-	8.74

**Table 3:** Chemical composition of *C. glomerata* [38].

Chemical Composition	Content of Chemical Composition
Carbohydrate	34.7 ± 0.4
C (wt.%)	31.33
O (wt.%)	30.67
Protein	26.3 ± 0.36
H (wt.%)	4.99
N (wt.%)	4.9
Lipid	2.4 ± 0.15
S (wt.%)	1.99

**Table 4:** Mineral metal content in *C. glomerata* [38].

Mineral Metal Content	Dry Mass (g·kg^−1^)
Potassium	94.1
Calcium	56.4
Iron	26.5
Aluminum	23.1
Magnesium	13.5
Sodium	11.5
Chrome	0.247

**Table 5:** Chemical compounds in *C. glomerata* [39].

Chemical Compounds	Content of Chemical Compounds (%)
n-Hexadecanoic acid (palmitic acid) (C16:0)	45.06
Hexanedioic acid, mono (2-ethylhexyl) ester	19.51
Tetradecanoic acid (myristic acid) (C14:0)	14.55
2–Pentadecanone, 6,10,14-trimethyl (C15:0)	10.53
Tetradecanoic acid, 12-methyl ester	4.48
Hexadecanoic acid, ethyl ester (ethyl palmitate)	2.92
9,12 Octadecadienoyl chloride (linoleoyl chloride)	2.92

**Table 6:** Elemental composition observed in the freshwater *L. minor* filaments.

Element	The Element Content (mg·kg^−1^)
Potassium	21,940
Calcium	13,020
Phosphorus	5411
Magnesium	2455
Nitrogen	874
Iron	485
Boron	446
Copper	328
Zinc	94.9
Manganese	68.4

**Table 7:** Elemental composition observed in the freshwater *C. glomerata* filaments in the summer of 2021.

Element	The Element Content (g∙g^−1^ of Dry Mass)
Calcium	148.2 ± 3.1
Potassium	18.24 ± 0.16
Magnesium	3.48 ± 0.02
Lead	0.75 ± 0.05
Arsenic	0.51 ± 0.04
Sodium	0.45 ± 0.02
Iron	0.21 ± 0.01
Nickel	0.12 ± 0.03
Manganese	0.08 ± 0.03
Cadmium	0.06 ± 0.01
Copper	0.05 ± 0.01
Zinc	0.03 ± 0.01
Cobalt	0.01 + 0.001
Chrome	0.01 + 0.001

**Table 8:** The mean value, the minimum value, the maximum value, and the standard deviation of the size of the patches of *Cladophora glomerata* and *Lemna minor* (m^3^).

Date	*Cladophora glomerata*			
	Mean	Minimum	Maximum	Standard Deviation
30 April 2021	0.015	0.01	0.02	0.005
14 June 2021	0.377	0.32	0.42	0.051
17 July 2021	0.700	0.65	0.76	0.056
	***Lemna minor***			
30 April 2021	0.15	0.10	0.20	0.050
14 June 2021	0.15	0.10	0.19	0.046
17 July 2021	0.09	0.08	0.10	0.010

**Table 9:** The mean value, the minimum value, the maximum value, and the standard deviation of phenol concentration of *C. glomerata* from Oporzyńskie Lake, *L. minor* from Oporzyńskie Lake, and *L. minor* from breeding (mg GAE g^−1^).

Date	*Cladophora glomerata*			
	Mean	Minimum	Maximum	Standard Deviation
30 April 2021	7.515	7.418	7.601	0.092
14 June 2021	18.209	17.934	18.459	0.263
17 July 2021	17.921	17.306	18.269	0.534
	***Lemna minor*** (from Oporzyńskie Lake)			
30 April 2021	2.412	2.111	2.892	0.420
14 June 2021	6.480	5.987	6.876	0.452
17 July 2021	9.582	9.423	9.816	0.201
	***Lemna minor*** (from breeding)			
30 April 2021	1.902	1.794	2.006	0.106
14 June 2021	2.035	2.014	2.052	0.019
17 July 2021	2.034	2.029	2.042	0.007

## Data Availability

Not applicable.

## Sample Availability

Samples of *Lemna minor* and *Cladophora glomerata* are available from the authors.
